# Safety and Accuracy of Suction Rectal Biopsy in Preterm Infants

**DOI:** 10.3389/fped.2021.642342

**Published:** 2021-08-20

**Authors:** Yanan Zhang, Yongwei Chen, Shen Yang, Yichao Gu, Kaiyun Hua, Yong Zhao, Jinshi Huang

**Affiliations:** Beijing Children's Hospital, Capital Medical University, National Center for Children's Health, Beijing, China

**Keywords:** suction rectal biopsy, Hirschsprung disease, preterm infants, safety, accuracy

## Abstract

**Purpose:** Most pediatric surgeons give little attention to the diagnosis of Hirschsprung disease (HD) in preterm infants. We aimed to explore the safety and accuracy of suction rectal biopsy (SRB) for diagnosing HD in preterm infants.

**Methods:** A retrospective review was conducted of 45 preterm patients who underwent SRB from 2015 to 2019 in our hospital. We collected the clinical characteristics and pathology results of the patients and information on follow-up. The sensitivity and specificity of SRB for HD diagnosis were calculated.

**Results:** The median gestational age of the patients was 35 weeks (range: 28.9–36.9 weeks), the median gestational age at biopsy was 38.6 weeks (range: 33.4–60.0 weeks), and the median weight was 2,790 g (range: 1,580–4,100 g). Fifteen patients (33.3%) were positive for HD, which was confirmed after pull-through surgery. Ganglion cells were present in 30 patients. The diagnosis of HD was excluded in 29 patients after discharge follow-up. The sensitivity of SRB ranged from 93.7 to 100%, and the specificity was 100%. No complications occurred after SRB among the patients whose biopsy age was <37 weeks (10 patients) or biopsy weight was <2,000 g (five patients).

**Conclusion:** SRB is accurate and safe for diagnosing HD in late preterm infants.

## Introduction

Hirschsprung disease (HD) in premature infants has attracted increasing attention, as preterm infants' medical conditions have improved in recent years. Delayed passage of meconium is common in premature infants, and the incidence appears to be inversely correlated with gestational age (1). In the past, the occurrence of HD in premature infants was considered uncommon; symptoms of delayed passage were attributed to the immature development of the enteric nervous system (2). A case series in 2013 described a cohort of premature newborns with HD treated at a single center and showed that HD occurs significantly less often in premature infants than in term infants; hence, suction rectal biopsy (SRB) should be used more selectively in preterm infants (3). However, in recent years, two large population-based studies have shown that preterm HD (PHD) is comparable to term infant HD in terms of occurrence. The incidences of both are approximately 1/5,000. In addition, PHD accounts for approximately 6% of all HD. The diagnosis of HD is often delayed in premature newborns (4, 5). From the perspective of embryonic development, it is currently believed that HD is caused by the destruction of the early embryonic neuroblast migration process. The migration of neuroblasts generally occurs between 7 and 12 weeks of gestation in human embryos (6, 7). Therefore, confirming PHD early is beneficial to clinicians so that appropriate treatment plans can be made and focus shifted to other diagnoses if a diagnosis of HD is excluded. SRB is considered the gold standard for the diagnosis of HD. However, it is often delayed until the child reaches term-adjusted gestational age due to the belief that SRB is unreliable in preterm infants. The objective of this study was to quantify the sensitivity, specificity, complications and outcomes associated with SRB in preterm infants by reviewing the data for our preterm infants suspected of having HD.

## Methods

### Patients

We performed a retrospective review of preterm infants (gestational age <37 weeks) who underwent SRB from December 2015 to June 2019. We selected and documented the following data: sex, gestational age of birth, gestational age of biopsy, weight of birth, weight of biopsy, biopsy results, procedural complications, surgical procedures performed, and clinical outcome.

### SRB Procedure

All SRBs were performed by the pediatric surgical fellow or attending surgeon. We used the standard techniques as described on the RBi2 website (8). Rectal irrigation was performed before the biopsy. The device with the cartridge was inserted into the anus to approximately 3 cm to 4 cm from the exit of the anal canal. At least 2 biopsies were performed posteriorly or laterally (6 o'clock or 4, 8 o'clock). Specimens were delivered to pathology in formalin. Both mucosal and submucosal layers were included in each biopsy.

The biopsies were processed and embedded using routine procedures. At least six slides were made from one tissue, with more being obtained for staining as available. Hematoxylin and eosin staining and calretinin immunohistochemical staining were performed with tissue from each patient. All pathology slides were reviewed by a pathologist with subspecialty training in pediatric pathology.

### Statistical Analysis

We analyzed all the data with SPSS 23.0. Continuous variables were presented as the mean with standard deviation or median and interquartile range if the normality hypothesis test rejected the null hypothesis of normal distribution. Categorical variables were reported as counts and percentages. The sensitivity and specificity of SRB for HD diagnosis were calculated.

## Results

### Patient Characteristics

Forty-five preterm infants underwent SRB during the study period. Table 1 presents the demographic information of the patients in the study cohort. Males comprised 48.9% of the cohort, while females comprised 51.1%. The median age at birth was 35 weeks (range: 28.9–36.9 weeks), and the median age at biopsy was 38.6 weeks (range: 33.4–60 weeks). The median weight at biopsy was 2,790 g (range: 1,580–4,100 g). No biopsy complications were found in our cases.

**Table 1:** Patient characteristics.

**Clinical characteristic**	***n* (%)**
**Sex**
Male	22 (48.9)
Female	23 (51.1)
**Age at birth**
<33 weeks	10 (22.2)
33–34 weeks	6 (13.3)
34–35 weeks	3 (6.7)
35–36 weeks	13 (28.9)
36–37 weeks	13 (28.9)
**Age at biopsy**
<33 weeks	0
33–34 weeks	2 (4.4)
34–35 weeks	1 (2.2)
35–36 weeks	3 (6.7)
36–37 weeks	4 (8.9)
37–40 weeks	18 (40.0)
>40 weeks	17 (37.8)
**Weight at biopsy**
<1,750 g	2 (4.4)
1,750–1,999 g	3 (6.7)
2,000–2,249 g	6 (13.3)
2,250–2,499 g	5 (11.1)
2,500–2,999 g	11 (24.4)
>3,000 g	18 (40.0)

### Pathological Features, Treatment Strategies, and Prognosis

As shown in Table 2, histologic examination reports were grouped into three types. One group of patients had normal, calretinin-positive ganglion cells in the submucosa. The HD patients lacked ganglion cells with hypertrophic nerves and were calretinin negative. The third group of patients had ganglion cells in the submucosa, but some of them were hypoplastic ganglion cells and were calretinin positive. All 15 patients whose biopsy specimens lacked ganglion cells underwent surgery, namely, a pull-through operation (*n* = 12), colostomy (*n* = 2), and ileostomy (*n* = 1). The colostomy patients underwent a pull-through operation after 3 months. The ileostomy patient was confirmed to have total colonic HD. The patient's parents abandoned further treatment, so this patient had no further follow-up records. The diagnosis of HD was confirmed through demonstration of an aganglionic segment on final pathology in all 15 patients. Among the 30 patients with ganglion cells on initial biopsy, the diagnosis of HD was excluded in 29 patients after discharge follow-up. The median follow-up period was 2 years (range: 1–4 years). Five of the patients underwent ileostomy because of necrotizing enterocolitis (NEC) or bowel resection for gut stenosis after NEC. Close surgeries were performed after 3 months. Symptoms resolved with appropriate treatment in each case. One patient died at home a few days after treatment was abandoned.

**Table 2:** Pathological features, treatment strategies, and prognosis of the patients.

**Pathological diagnosis**	**Treatment strategies**	**Outcome**
HD (*n* = 15)	Pull-through surgery (*n* = 12); Colostomy 2; Ileostomy (total colonic, n = 1)	Normal^a^
Normal (*n* = 22)	No surgery (*n* = 17); Surgery (NEC or gut stenosis after NEC, *n* = 5)	Normal
Hypoplasia ganglion cell partly (else normal, *n* = 8)	No surgery (*n* = 8)	One died^b^, the rest were normal

a *Well-developed, no gastrointestinal symptoms*.

b *The baby's parents abandoned the treatment and went home*.

Among the 15 PHD patients (10 boys and 5 girls), 8 had rectosigmoid disease, 6 had long segment disease, and 1 had total colonic aganglionosis. The median gestational age at the operation was 38.6 weeks (range: 36.3 to 43.4 weeks), and the median weight was 2,940 g (range: 2,100 to 4,000 g). Among all the patients, 4 had a weight of <2,500 g.

### The Accuracy of SRB in Preterm Infants

If we consider the patient who died after treatment abandonment whose biopsy result excluded a diagnosis of HD as a false negative since no autopsy was performed, the sensitivity of SRB was 93.7% (95% CI 67.7–99.7%), and the specificity was 100% (95% CI 85.4–100.0%) in the cohort. If the case was a true negative, the sensitivity of SRB was 100% (95% CI 74.7–99.7%), and the specificity was 100% (95% CI 85.9–100.0%).

## Discussion

SRB has proven to be a valuable diagnostic technique for HD since 1965, especially due to its high accuracy and minimal invasion. In recent years, it has been proven that the sensitivity and specificity of SRB are 96.8 and 99.4%, respectively, in some systematic reviews (9). SRBs are generally safe, and the most common complication is inadequate histology (10). However, another study reported that SRB and full-thickness rectal biopsy appear equivalent in their ability to provide adequate submucosa (11). The sensitivity and specificity of SRB in term-corrected infants has ranged from 46–100% and from 97–100%, respectively (12–14). Most clinicians agree that preterm patients who are suspected of having HD should not undergo SRB until reaching term-corrected age or gaining more weight. It has been confirmed that the intestinal wall muscle layer increases with age and that the intestinal wall of preterm infants is thinner than that of term infants. Therefore, in theory, the risk of bowel perforation in preterm infants undergoing rectal biopsy is greater than that of full-term or older children. Drs Meinds and Kuiper indicated that infant age influenced the accuracy of SRBs for diagnosing HD. SRB was used to identify HD in patients younger than 39 days old with significantly lower sensitivity than in older patients (50 vs. 88%). The specificity with which SRB identified infants without HD was not affected by age (average 95%) (14). However, Halleran et al. reviewed the SRB of PHD at their institute and indicated that this procedure can be performed safely in preterm infants as small as 1,590–2,000 g with high accuracy. Clinicians should not hesitate to perform a biopsy for a premature infant when clinically appropriate (15). Keyzer-Dekker found that SRB can also be reliably and safely performed in preterm-born infants. The sensitivity and specificity of SRB were 83 and 97%, respectively (13). In our cases, 10 patients were younger than 37 gestational weeks at the time of biopsy. The biopsy results suggested HD in four of them, which was confirmed after surgery. Among the remaining patients, five were cured with appropriate treatment and have developed well to date. A total of 35 patients were older than 37 gestational weeks at the time of biopsy; among them, eleven had HD, which was verified during surgery. The remaining 24 cases were cured with appropriate treatment and have developed well to date. The sensitivity of SRB ranged from 93.7 to 100%, and the specificity was 100% in our cohort.

There were a total of 16 preterm infants whose biopsy weights were <2,500 g and five infants whose biopsy weights were <2,000 g. The youngest baby was 31.5 weeks old, with a biopsy age of 33.5 weeks, and his biopsy weight was 1,580 g. His biopsy results were normal. However, it is worth noting the safety of biopsy in premature infants because of their hypoplasia. In this research, the patients were a highly selected group with a stable condition and a high pretest probability of HD. This may be one reason why we have a small cohort. In addition, before SRB for the preterm infants, we performed the procedure for the term infants hundreds of times. It is more appropriate to use a 20 ml syringe as the suction device, and pulling the piston to 6 ml is the best position for most preterm cases based on our experience. Bleeding was defined as the absence of hemorrhage <2 ml. There were no complications in the cohort. In fact, there were some complications, either bleeding or inadequacy, in our past SRB data for term infants. Very few of them needed blood transfusion therapy. The incidence of that was approximately 1/200–300. That experience ensured that we performed better for the preterm infants in the current cohort. RSBs were performed when most of the patients reached term gestational age which was related to the concept of conservative treatment (the RSB was invasive). That might be one of the reasons for the good outcomes. At the same time, nearly 40% of these infants weigh <2,500 g and the risk was similar to that of premature infants. This study demonstrates that SRB can be used to reliably diagnose PHD. Because most premature infants have delayed feces, which is a symptom similar to the manifestations of HD, it is difficult to distinguish them. The accuracy of contrast was lower in younger HD patients, while the risk of NEC may increase. From our case, the safety and accuracy of SRB in late preterm infants were high. Early diagnosis can provide the best treatment for children in a targeted manner.

Based on the results of this study, we consider that performing SRB in premature infants with a biopsy age of older than 33 weeks is safe and accurate. Of course, more cases are needed to verify these conclusions. Investigators should evaluate the condition of the preterm infant. Bedside SRB is not universally recommended for premature infants <32 weeks old, especially those with a body weight of <1,500 g.

In addition, an experienced pathologist is very important. Immature ganglion cells may not be detected since the biopsy result is dependent on the entire process—material extraction, sectioning, staining, etc. Even if our clinicians obtain satisfactory specimens, namely, from the right region of the rectum (not taken too close to the dentate line) and of the appropriate size (at least 3 mm in diameter, and a minimum of one-third of the sample should include the submucosa according to the International Working Group of the 2009 World Congress of Gastroenterology), biopsies do not have ganglion cells on every slide. Our hospital's pathologists found that ~6–15 slides made from each tissue were enough, while some needed to be sliced continuously or repeatedly according to the specimen. Calretinin immunohistochemical staining was also performed, and ganglion cells were identified when both the nucleus and cytoplasm were calretinin positive. However, dysplastic ganglion cells show less typical staining than normal ganglion cells, and there is no uniform definition of the developmental stages of ganglion cells, such as cell size and nucleoplasm morphology. An experienced pathologist therefore has higher accuracy when identifying those cells. According to the follow-up results of our cases, the pathological results were consistent with the clinical outcomes.

Finally, we aimed to explore the safety and accuracy of SRB in preterm infants rather than the outcomes of premature infants suspected of having HD. The patients in this study were premature infants who had distention, vomiting or other intestinal symptoms. We chose these cases retrospectively because SRB was performed. There are a number of limitations to this small, retrospective study that limit its use in a broader population. While the results are encouraging that SRB can be performed early, the complications of concern occur very infrequently, and thus, a study of this size is underpowered to identify their true incidence. For the various clinical symptoms of premature infants, the timing of biopsy still needs to be determined by clinicians.

## Data Availability Statement

The original contributions presented in the study are included in the article/supplementary material, further inquiries can be directed to the corresponding author/s.

## Ethics Statement

The studies involving human participants were reviewed and approved by the Medical Ethics Committee of Beijing Children's Hospital (2020-Z-082).

## Author Contributions

YG, KH, YoZ, and YaZ participated in the clinical work. YaZ carried out data collection, analysis of data and preparation of the manuscript. YC and JH designed the study. SY participated in the analysis of data and preparation of the manuscript. All authors read and approved the final manuscript.

## Conflict of Interest

The authors declare that the research was conducted in the absence of any commercial or financial relationships that could be construed as a potential conflict of interest.

## Publisher's Note

All claims expressed in this article are solely those of the authors and do not necessarily represent those of their affiliated organizations, or those of the publisher, the editors and the reviewers. Any product that may be evaluated in this article, or claim that may be made by its manufacturer, is not guaranteed or endorsed by the publisher.
